# A case of a large venous ring around the mandibular condyle

**DOI:** 10.1007/s00276-025-03602-2

**Published:** 2025-03-11

**Authors:** Keitaro Nishi, Tatsuo Okui, Yohei Takeshita, Jingo Kusukawa, R. Shane Tubbs, Joe Iwanaga

**Affiliations:** 1 Department of Maxillofacial Diagnostic and Surgical Science, Field of Oral and Maxillofacial Rehabilitation, Graduate School of Medical and Dental Sciences, Kagoshima University, Kagoshima, 890-8544 Japan; 2 Department of Oral and Maxillofacial Radiology, Faculty of Medicine, Dentistry and Pharmaceutical Sciences, Okayama University, Okayama, 700-8558 Japan; 3 Dental and Oral Medical Center, Kurume University School of Medicine, 67 Asahi-machi, Kurume, Fukuoka 830-0011 Japan; 4 Department of Neurosurgery, Clinical Neuroscience Research Center, Tulane University School of Medicine, 131 S. Robertson St., Suite 1300, New Orleans, LA 70112 USA; 5 Department of Neurology, Clinical Neuroscience Research Center, Tulane University School of Medicine, New Orleans, LA USA; 6 Department of Structural and Cellular Biology, Tulane University School of Medicine, New Orleans, LA USA; 7 Department of Neurosurgery and Ochsner Neuroscience Institute, Ochsner Health System, New Orleans, LA USA; 8 Department of Anatomical Sciences, St. George’s University, St. George’s, Grenada; 9 Department of Surgery, Tulane University School of Medicine, New Orleans, LA USA; 10 University of Queensland, Brisbane, Australia; 11 Division of Gross and Clinical Anatomy, Department of Anatomy, Kurume University School of Medicine, 67 Asahi-machi, Kurume, Fukuoka Japan

**Keywords:** Maxillary vein, Temporomandibular joint, Cadaver, Anatomy

## Abstract

Anatomical details regarding venous drainage of the head and neck are an important matter for surgeons to avoid unnecessary complications such as hemorrhage. This report describes a case of the large venous ring around the mandibular condyle found in the cadaver. The left maxillofacial region of a latex-injected embalmed male cadaver (82 years of age at death) was dissected. The large two maxillary veins ran lateral to the capsule and superior to the mandibular notch and coursed posteroinferiorly to merge, and one trunk was formed at the posterior border of the ramus. It then received the superficial temporal vein superiorly to form the retromandibular vein (RMV). In addition, three maxillary veins were drained from the pterygoid venous plexus (PVP), medial to the ramus, one maxillary vein drained from the PVP into the RMV trunk, while two maxillary veins drained from the PVP into the anterior division of the RMV. All five large veins lateral and medial to the condyle drained from the PVP into the RMV. The knowledge of such an anatomical variation might prevent intraoperative bleeding in the temporomandibular joint region.

## Introduction

The maxillary veins are formed by the confluence of the pterygoid venous plexus (PVP), run medial to the mandibular ramus posteriorly to join the superficial temporal vein, then form the retromandibular vein (RMV) [10]. The maxillary veins have often been overlooked in the literature due to their deep location, although the variations of the RMV and external jugular veins have been extensively reported [2, 11]. Generally, the maxillary vein runs medial to the mandibular ramus with the maxillary artery (MA) [3], which is often discussed during orthognathic surgeries such as the bilateral sagittal split osteotomy (BSSO) [9]. Accurate anatomical knowledge of the maxillary veins is essential to reduce intraoperative bleeding [3].

For temporomandibular joint (TMJ) surgery, the nerves, such as the temporal branch of the facial nerve, and arteries, such as superficial and middle temporal arteries, are always focused [8], but not the veins, as the major veins are not shown lateral to the condyle in anatomy textbooks except for the transverse facial vein. If there are any major veins in that region due to the anatomical variation, the TMJ surgery could be more challenging. In this report, we describe a case of the variation of the maxillary veins and discuss its clinical relevance.

### Case presentation

**Fig. 1:**
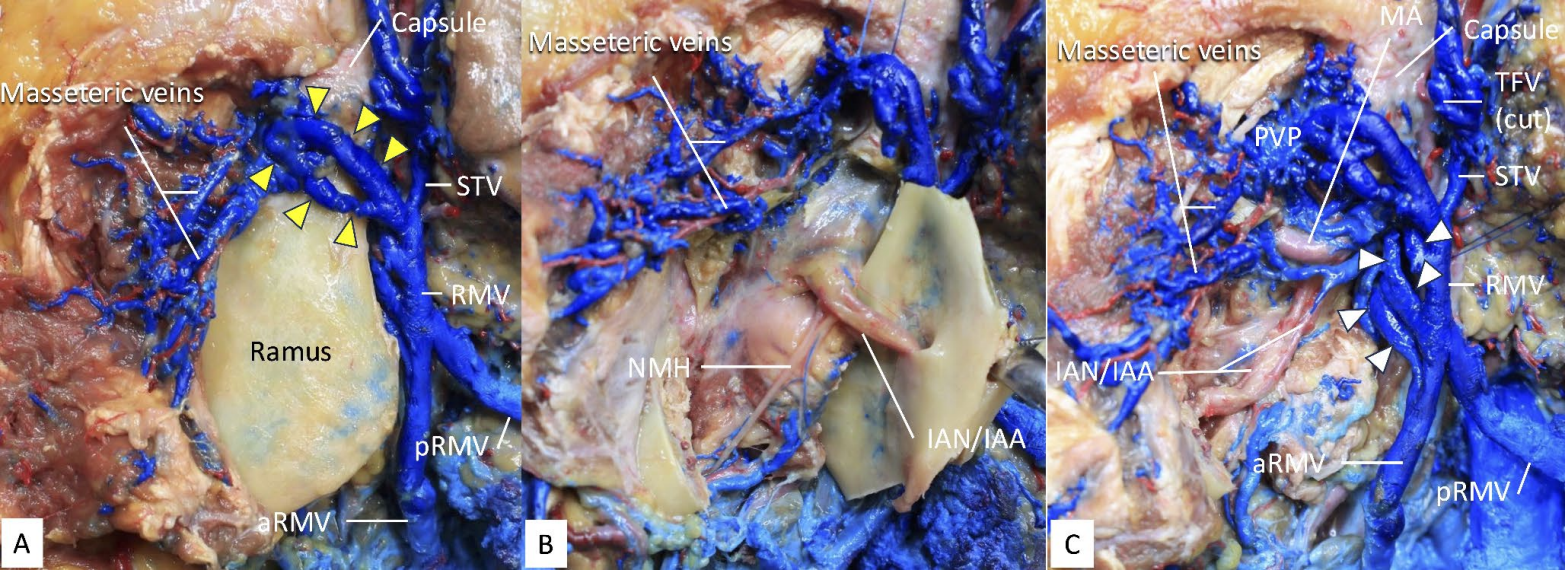
Variant maxillary veins lateral to the left mandibular condyle. (**A**) Two large maxillary veins (arrowheads) were found lateral to the left mandibular condyle. They merge into one trunk and join the superficial temporal vein (STV) to form the retromandibular vein (RMV). The RMV then split into anterior (aRMV) and posterior (pRMV) divisions. (**B**) The incised ramus was turned posteriorly. Note the ramus was detached from the medial periosteum. IAN/IAA, inferior alveolar nerve/artery. NMH, nerve to mylohyoid muscle. (**C**) After removing the ramus and medial periosteum, the pterygomandibular space is exposed. Note the three maxillary veins (arrowheads) were identified. IAN/IAA, inferior alveolar nerve/artery. MA, maxillary artery. PVP, pterygoid venous plexus. aRMV, anterior division of retromandibular vein. pRMV, posterior division of retromandibular vein. STV, superficial temporal vein. TFV, transverse facial vein

**Fig. 2:**
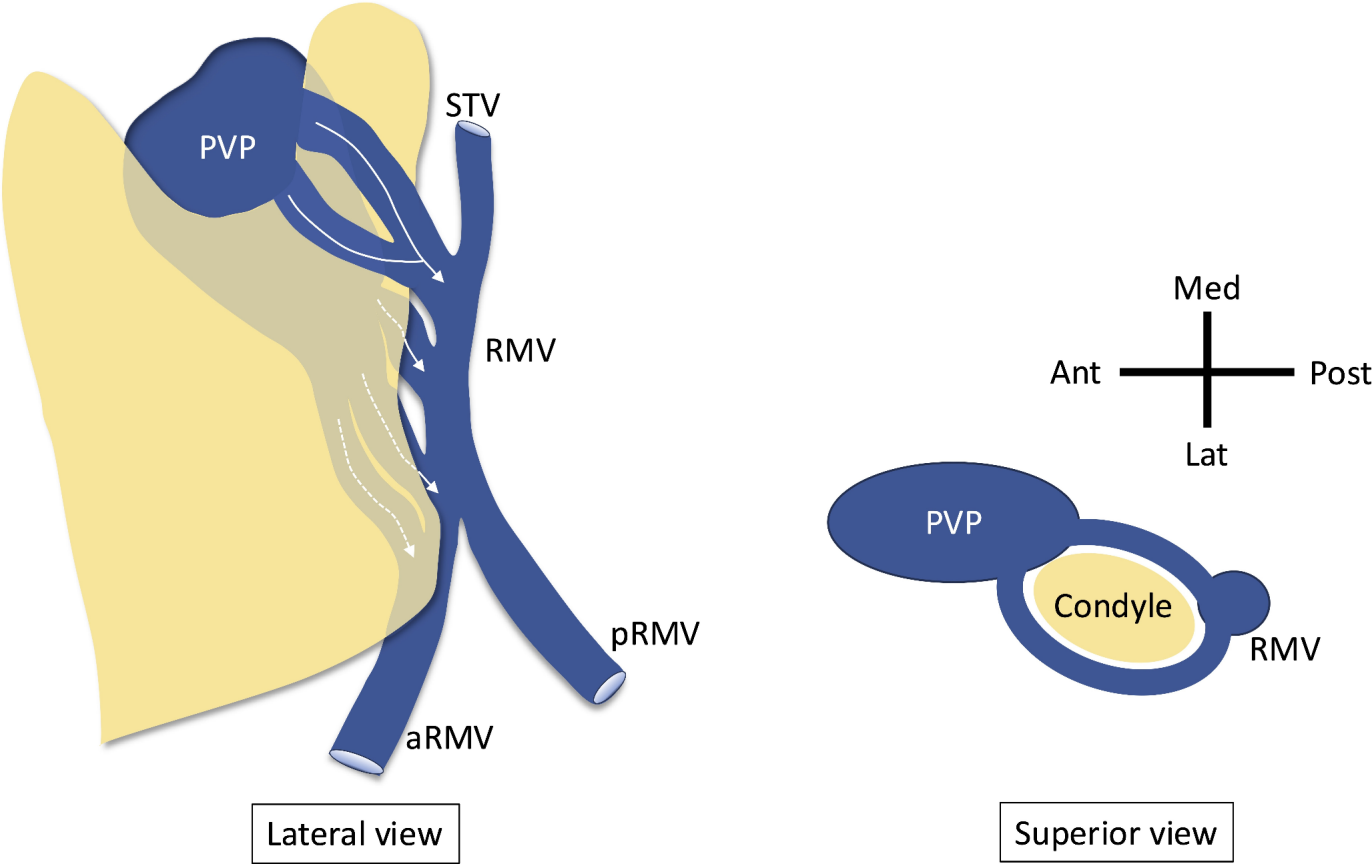
Schematic drawing of the pericondylar venous ring formed by the variant maxillary veins (curved arrows), pterygoid venous plexus (PVP), and retromandibular vein (RMV). aRMV, anterior division of retromandibular vein. pRMV, posterior division of retromandibular vein. STV, superficial temporal vein

During routine dissection of a latex-injected male cadaver, the age at the time of death, was 82 years old who did not have any history of facial trauma or obvious pathology, two large variant veins were found lateral to the left mandibular condyle (Fig. 1A). The veins were traveling outside the capsule and superior to the mandibular notch and coursed posteroinferiorly to merge and form one trunk at the posterior border of the ramus. The diameters of the superior one, inferior one, and trunk were 7.5 mm, 5.2 mm, and 8.3 mm, respectively. Then, it received the superficial temporal vein (STV) superiorly to form the RMV. The ramus was incised by a bone saw at the neck of the condyle, base of the coronoid process, and junction of the ramus and body of the mandible. The ramus was detached from the medial periosteum (Fig. 1B). To completely remove the ramus, the inferior alveolar nerve and artery were incised. After removing the ramus, the periosteum was removed to observe the pterygomandibular space. It was found that three large veins drained from the PVP into the RMV in the pterygomandibular space (Fig. 1C). One maxillary vein drained from the PVP into the RMV trunk, while two maxillary veins drained from the PVP into the anterior division of the RMV. The diameters of the superior, middle, and inferior ones were 5.1 mm, 5.9 mm, and 5.0 mm, respectively. The masseteric veins were observed draining into the PVP through the mandibular notch, anterior to the maxillary veins, with the masseter muscle retracted anteriorly. The transverse facial vein was severed at its junction with the STV. With all five large veins lateral and medial to the condyle draining from the PVP into RMV, we named this variant venous system “pericondylar venous ring (Fig. 2).” To our knowledge, such an anatomical variation of the maxillary veins has not been reported previously.

The maxillary and retromandibular veins on the right side did not vary. The authors state that every effort was made to follow all local and international ethical guidelines and laws regarding the use of human cadaveric donors in anatomical research.

## Discussion

Development of the head and neck veins begins on embryonic day 27. The primitive maxillary vein is the second venous tract in development, located cephalad and lateral to the ventral pharyngeal vein. The primitive maxillary vein branches extensively at the emerging maxillary process and eventually drains into the trigeminal nerve area. The first major drainage source of the maxillary vein is the developing eye, but it ultimately becomes the origin of all ophthalmic and orbital veins in adults. At approximately embryonic day 50, the development of the primitive maxillary vein reaches its uppermost point. Therefore, the primitive maxillary vein begins to extend cephalad and caudal to the area innervated by the other two branches of the trigeminal nerve. By embryonic day 54, This development begins the formation of the prospective anterior facial vein, and the primitive maxillary vein eventually joins the linguofacial vein in the upper part of the mandible to become the common facial vein, which merges with the RMV and drains through the linguofacial vein to the internal jugular vein [4, 11].

Several variations of the maxillary veins have been reported. Wang’s case presented that the RMV was bypassed and joined directly to the facial vein [11]. Patil et al. (2014) reported the absence of RMV, which presented two maxillary veins running anteriorly and posteriorly, forming an anomalous external jugular vein [5]. Manta et al. (2021) found venous variation, which shows communication between the maxillary and external jugular veins that resulted in the circumflex vein. The authors called the vein lateral to the condyle “extracondylar vein” [2]. The present case demonstrated two large veins lateral to the condyle (similar to the extracondylar vein by Manta et al.), which became one trunk before reaching the RMV, and three large maxillary veins drained into the RMV. All five large veins, lateral and medial to the condyle draining from the PVP into RMV, can be damaged by any TMJ-related or ramus-related surgical procedures.We named this variant venous system the “pericondylar venous ring.” In 1903, Poirier described the similar venous plexus [6]. In our observation, there are only a couple of abnormal veins lateral to the condyle that drain into the RMV. The only structure resembling a plexus in this case was the PVP. Additionally, a venous plexus is typically a collection of numerous small veins, rather than just a few large veins. Based on these findings, we prefer to describe this structure as a “venous ring.” It is generally considered intraoperative injury of the maxillary artery and the inferior alveolar nerve can be avoided by separating the periosteum from the bone [7]. However, arterial bleeding is a well-recognized complication of the preauricular approach to the TMJ, as there is no protection such as the periosteum [1]. The pericondylar venous ring could increase the risk of intraoperative bleeding for the same reason during any procedure related to the TMJ and ramus. Although this should be extremely rare, surgeons must be aware of this anatomical variation.

## Conclusions

Surgeons must be knowledgeable about the anatomical variations of the venous system around the TMJ. Venous variations can be identified during preoperative diagnosis using magnetic resonance imaging (MRI) or contrast CT.

## Data Availability

No datasets were generated or analysed during the current study.
